# Supplementary material for: OpenMM 7: Rapid development of high performance algorithms for molecular dynamics
Source: PLoS Comput Biol. 2017 Jul 26;13(7):e1005659. doi: 10.1371/journal.pcbi.1005659 (PMC5549999; doi:10.1371/journal.pcbi.1005659)
Supplement: S1 Source Code — (ZIP) [file pcbi.1005659.s002.zip › OpenMM-7.0.1-Source/docs/api-c++/generated/OpenMM.NonbondedForce.html]

NonbondedForce — OpenMM @OPENMM\_MAJOR\_VERSION@.@OPENMM\_MINOR\_VERSION@ documentation


# NonbondedForce¶

*class* `OpenMM::``NonbondedForce`¶
:   This class implements nonbonded interactions between particles, including a Coulomb force to represent electrostatics and a Lennard-Jones force to represent van der Waals interactions. It optionally supports periodic boundary conditions and cutoffs for long range interactions. Lennard-Jones interactions are calculated with the Lorentz-Berthelot combining rule: it uses the arithmetic mean of the sigmas and the geometric mean of the epsilons for the two interacting particles.

    To use this class, create a `NonbondedForce` object, then call `addParticle()` once for each particle in the `System` to define its parameters. The number of particles for which you define nonbonded parameters must be exactly equal to the number of particles in the `System`, or else an exception will be thrown when you try to create a `Context`. After a particle has been added, you can modify its force field parameters by calling `setParticleParameters()`. This will have no effect on Contexts that already exist unless you call `updateParametersInContext()`.
    `NonbondedForce` also lets you specify “exceptions”, particular pairs of particles whose interactions should be computed based on different parameters than those defined for the individual particles. This can be used to completely exclude certain interactions from the force calculation, or to alter how they interact with each other.

    Many molecular force fields omit Coulomb and Lennard-Jones interactions between particles separated by one or two bonds, while using modified parameters for those separated by three bonds (known as “1-4 interactions”). This class provides a convenience method for this case called `createExceptionsFromBonds()`. You pass to it a list of bonds and the scale factors to use for 1-4 interactions. It identifies all pairs of particles which are separated by 1, 2, or 3 bonds, then automatically creates exceptions for them.

    When using a cutoff, by default Lennard-Jones interactions are sharply truncated at the cutoff distance. Optionally you can instead use a switching function to make the interaction smoothly go to zero over a finite distance range. To enable this, call `setUseSwitchingFunction()`. You must also call `setSwitchingDistance()` to specify the distance at which the interaction should begin to decrease. The switching distance must be less than the cutoff distance.

    Another optional feature of this class (enabled by default) is to add a contribution to the energy which approximates the effect of all Lennard-Jones interactions beyond the cutoff in a periodic system. When running a simulation at constant pressure, this can improve the quality of the result. Call `setUseDispersionCorrection()` to set whether this should be used.

    Methods

    |  |  |
    | --- | --- |
    | `NonbondedForce` | Create a `NonbondedForce`. |
    | `getNumParticles` | Get the number of particles for which force field parameters have been defined. |
    | `getNumExceptions` | Get the number of special interactions that should be calculated differently from other interactions. |
    | `getNonbondedMethod` | Get the method used for handling long range nonbonded interactions. |
    | `setNonbondedMethod` | Set the method used for handling long range nonbonded interactions. |
    | `getCutoffDistance` | Get the cutoff distance (in nm) being used for nonbonded interactions. |
    | `setCutoffDistance` | Set the cutoff distance (in nm) being used for nonbonded interactions. |
    | `getUseSwitchingFunction` | Get whether a switching function is applied to the Lennard-Jones interaction. |
    | `setUseSwitchingFunction` | Set whether a switching function is applied to the Lennard-Jones interaction. |
    | `getSwitchingDistance` | Get the distance at which the switching function begins to reduce the Lennard-Jones interaction. |
    | `setSwitchingDistance` | Set the distance at which the switching function begins to reduce the Lennard-Jones interaction. |
    | `getReactionFieldDielectric` | Get the dielectric constant to use for the solvent in the reaction field approximation. |
    | `setReactionFieldDielectric` | Set the dielectric constant to use for the solvent in the reaction field approximation. |
    | `getEwaldErrorTolerance` | Get the error tolerance for Ewald summation. |
    | `setEwaldErrorTolerance` | Set the error tolerance for Ewald summation. |
    | `getPMEParameters` | Get the parameters to use for PME calculations. |
    | `setPMEParameters` | Set the parameters to use for PME calculations. |
    | `getPMEParametersInContext` | Get the parameters being used for PME in a particular `Context`. |
    | `addParticle` | Add the nonbonded force parameters for a particle. |
    | `getParticleParameters` | Get the nonbonded force parameters for a particle. |
    | `setParticleParameters` | Set the nonbonded force parameters for a particle. |
    | `addException` | Add an interaction to the list of exceptions that should be calculated differently from other interactions. |
    | `getExceptionParameters` | Get the force field parameters for an interaction that should be calculated differently from others. |
    | `setExceptionParameters` | Set the force field parameters for an interaction that should be calculated differently from others. |
    | `createExceptionsFromBonds` | Identify exceptions based on the molecular topology. |
    | `getUseDispersionCorrection` | Get whether to add a contribution to the energy that approximately represents the effect of Lennard-Jones interactions beyond the cutoff distance. |
    | `setUseDispersionCorrection` | Set whether to add a contribution to the energy that approximately represents the effect of Lennard-Jones interactions beyond the cutoff distance. |
    | `getReciprocalSpaceForceGroup` | Get the force group that reciprocal space interactions for Ewald or PME are included in. |
    | `setReciprocalSpaceForceGroup` | Set the force group that reciprocal space interactions for Ewald or PME are included in. |
    | `updateParametersInContext` | Update the particle and exception parameters in a `Context` to match those stored in this `Force` object. |
    | `usesPeriodicBoundaryConditions` | Returns whether or not this force makes use of periodic boundary conditions. |

    Enum: NonbondedMethod

    |  |  |
    | --- | --- |
    | **NoCutoff** | No cutoff is applied to nonbonded interactions. The full set of N^2 interactions is computed exactly. This necessarily means that periodic boundary conditions cannot be used. This is the default. |
    | **CutoffNonPeriodic** | Interactions beyond the cutoff distance are ignored. Coulomb interactions closer than the cutoff distance are modified using the reaction field method. |
    | **CutoffPeriodic** | Periodic boundary conditions are used, so that each particle interacts only with the nearest periodic copy of each other particle. Interactions beyond the cutoff distance are ignored. Coulomb interactions closer than the cutoff distance are modified using the reaction field method. |
    | **Ewald** | Periodic boundary conditions are used, and Ewald summation is used to compute the interaction of each particle with all periodic copies of every other particle. |
    | **PME** | Periodic boundary conditions are used, and Particle-Mesh Ewald (PME) summation is used to compute the interaction of each particle with all periodic copies of every other particle. |

    `NonbondedForce`()¶
    :   Create a `NonbondedForce`.

    int `getNumParticles`() *const*¶
    :   Get the number of particles for which force field parameters have been defined.

    int `getNumExceptions`() *const*¶
    :   Get the number of special interactions that should be calculated differently from other interactions.

    NonbondedMethod `getNonbondedMethod`() *const*¶
    :   Get the method used for handling long range nonbonded interactions.

    void `setNonbondedMethod`(NonbondedMethod *method*)¶
    :   Set the method used for handling long range nonbonded interactions.

    double `getCutoffDistance`() *const*¶
    :   Get the cutoff distance (in nm) being used for nonbonded interactions. If the NonbondedMethod in use is NoCutoff, this value will have no effect.

        |  |  |
        | --- | --- |
        | Returns: | the cutoff distance, measured in nm |

    void `setCutoffDistance`(double *distance*)¶
    :   Set the cutoff distance (in nm) being used for nonbonded interactions. If the NonbondedMethod in use is NoCutoff, this value will have no effect.

        |  |  |
        | --- | --- |
        | Parameters: |  |

        - **distance** – the cutoff distance, measured in nm

    bool `getUseSwitchingFunction`() *const*¶
    :   Get whether a switching function is applied to the Lennard-Jones interaction. If the nonbonded method is set to NoCutoff, this option is ignored.

    void `setUseSwitchingFunction`(bool *use*)¶
    :   Set whether a switching function is applied to the Lennard-Jones interaction. If the nonbonded method is set to NoCutoff, this option is ignored.

    double `getSwitchingDistance`() *const*¶
    :   Get the distance at which the switching function begins to reduce the Lennard-Jones interaction. This must be less than the cutoff distance.

    void `setSwitchingDistance`(double *distance*)¶
    :   Set the distance at which the switching function begins to reduce the Lennard-Jones interaction. This must be less than the cutoff distance.

    double `getReactionFieldDielectric`() *const*¶
    :   Get the dielectric constant to use for the solvent in the reaction field approximation.

    void `setReactionFieldDielectric`(double *dielectric*)¶
    :   Set the dielectric constant to use for the solvent in the reaction field approximation.

    double `getEwaldErrorTolerance`() *const*¶
    :   Get the error tolerance for Ewald summation. This corresponds to the fractional error in the forces which is acceptable. This value is used to select the reciprocal space cutoff and separation parameter so that the average error level will be less than the tolerance. There is not a rigorous guarantee that all forces on all atoms will be less than the tolerance, however.

        For PME calculations, if `setPMEParameters()` is used to set alpha to something other than 0, this value is ignored.

    void `setEwaldErrorTolerance`(double *tol*)¶
    :   Set the error tolerance for Ewald summation. This corresponds to the fractional error in the forces which is acceptable. This value is used to select the reciprocal space cutoff and separation parameter so that the average error level will be less than the tolerance. There is not a rigorous guarantee that all forces on all atoms will be less than the tolerance, however.

        For PME calculations, if `setPMEParameters()` is used to set alpha to something other than 0, this value is ignored.

    void `getPMEParameters`(double &*alpha*, int &*nx*, int &*ny*, int &*nz*) *const*¶
    :   Get the parameters to use for PME calculations. If alpha is 0 (the default), these parameters are ignored and instead their values are chosen based on the Ewald error tolerance.

        |  |  |
        | --- | --- |
        | Parameters: |  |

        - **alpha** – [out] the separation parameter
        - **nx** – [out] the number of grid points along the X axis
        - **ny** – [out] the number of grid points along the Y axis
        - **nz** – [out] the number of grid points along the Z axis

    void `setPMEParameters`(double *alpha*, int *nx*, int *ny*, int *nz*)¶
    :   Set the parameters to use for PME calculations. If alpha is 0 (the default), these parameters are ignored and instead their values are chosen based on the Ewald error tolerance.

        |  |  |
        | --- | --- |
        | Parameters: |  |

        - **alpha** – the separation parameter
        - **nx** – the number of grid points along the X axis
        - **ny** – the number of grid points along the Y axis
        - **nz** – the number of grid points along the Z axis

    void `getPMEParametersInContext`(*const* Context &*context*, double &*alpha*, int &*nx*, int &*ny*, int &*nz*) *const*¶
    :   Get the parameters being used for PME in a particular `Context`. Because some platforms have restrictions on the allowed grid sizes, the values that are actually used may be slightly different from those specified with `setPMEParameters()`, or the standard values calculated based on the Ewald error tolerance. See the manual for details.

        |  |  |
        | --- | --- |
        | Parameters: |  |

        - **context** – the `Context` for which to get the parameters
        - **alpha** – [out] the separation parameter
        - **nx** – [out] the number of grid points along the X axis
        - **ny** – [out] the number of grid points along the Y axis
        - **nz** – [out] the number of grid points along the Z axis

    int `addParticle`(double *charge*, double *sigma*, double *epsilon*)¶
    :   Add the nonbonded force parameters for a particle. This should be called once for each particle in the `System`. When it is called for the i’th time, it specifies the parameters for the i’th particle. For calculating the Lennard-Jones interaction between two particles, the arithmetic mean of the sigmas and the geometric mean of the epsilons for the two interacting particles is used (the Lorentz-Berthelot combining rule).

        |  |  |
        | --- | --- |
        | Parameters: |  |

        - **charge** – the charge of the particle, measured in units of the proton charge
        - **sigma** – the sigma parameter of the Lennard-Jones potential (corresponding to the van der Waals radius of the particle), measured in nm
        - **epsilon** – the epsilon parameter of the Lennard-Jones potential (corresponding to the well depth of the van der Waals interaction), measured in kJ/mol

        |  |  |
        | --- | --- |
        | Returns: | the index of the particle that was added |

    void `getParticleParameters`(int *index*, double &*charge*, double &*sigma*, double &*epsilon*) *const*¶
    :   Get the nonbonded force parameters for a particle.

        |  |  |
        | --- | --- |
        | Parameters: |  |

        - **index** – the index of the particle for which to get parameters
        - **charge** – [out] the charge of the particle, measured in units of the proton charge
        - **sigma** – [out] the sigma parameter of the Lennard-Jones potential (corresponding to the van der Waals radius of the particle), measured in nm
        - **epsilon** – [out] the epsilon parameter of the Lennard-Jones potential (corresponding to the well depth of the van der Waals interaction), measured in kJ/mol

    void `setParticleParameters`(int *index*, double *charge*, double *sigma*, double *epsilon*)¶
    :   Set the nonbonded force parameters for a particle. When calculating the Lennard-Jones interaction between two particles, it uses the arithmetic mean of the sigmas and the geometric mean of the epsilons for the two interacting particles (the Lorentz-Berthelot combining rule).

        |  |  |
        | --- | --- |
        | Parameters: |  |

        - **index** – the index of the particle for which to set parameters
        - **charge** – the charge of the particle, measured in units of the proton charge
        - **sigma** – the sigma parameter of the Lennard-Jones potential (corresponding to the van der Waals radius of the particle), measured in nm
        - **epsilon** – the epsilon parameter of the Lennard-Jones potential (corresponding to the well depth of the van der Waals interaction), measured in kJ/mol

    int `addException`(int *particle1*, int *particle2*, double *chargeProd*, double *sigma*, double *epsilon*, bool *replace* = false)¶
    :   Add an interaction to the list of exceptions that should be calculated differently from other interactions. If chargeProd and epsilon are both equal to 0, this will cause the interaction to be completely omitted from force and energy calculations.

        In many cases, you can use `createExceptionsFromBonds()` rather than adding each exception explicitly.

        |  |  |
        | --- | --- |
        | Parameters: |  |

        - **particle1** – the index of the first particle involved in the interaction
        - **particle2** – the index of the second particle involved in the interaction
        - **chargeProd** – the scaled product of the atomic charges (i.e. the strength of the Coulomb interaction), measured in units of the proton charge squared
        - **sigma** – the sigma parameter of the Lennard-Jones potential (corresponding to the van der Waals radius of the particle), measured in nm
        - **epsilon** – the epsilon parameter of the Lennard-Jones potential (corresponding to the well depth of the van der Waals interaction), measured in kJ/mol
        - **replace** – determines the behavior if there is already an exception for the same two particles. If true, the existing one is replaced. If false, an exception is thrown.

        |  |  |
        | --- | --- |
        | Returns: | the index of the exception that was added |

    void `getExceptionParameters`(int *index*, int &*particle1*, int &*particle2*, double &*chargeProd*, double &*sigma*, double &*epsilon*) *const*¶
    :   Get the force field parameters for an interaction that should be calculated differently from others.

        |  |  |
        | --- | --- |
        | Parameters: |  |

        - **index** – the index of the interaction for which to get parameters
        - **particle1** – [out] the index of the first particle involved in the interaction
        - **particle2** – [out] the index of the second particle involved in the interaction
        - **chargeProd** – [out] the scaled product of the atomic charges (i.e. the strength of the Coulomb interaction), measured in units of the proton charge squared
        - **sigma** – [out] the sigma parameter of the Lennard-Jones potential (corresponding to the van der Waals radius of the particle), measured in nm
        - **epsilon** – [out] the epsilon parameter of the Lennard-Jones potential (corresponding to the well depth of the van der Waals interaction), measured in kJ/mol

    void `setExceptionParameters`(int *index*, int *particle1*, int *particle2*, double *chargeProd*, double *sigma*, double *epsilon*)¶
    :   Set the force field parameters for an interaction that should be calculated differently from others. If chargeProd and epsilon are both equal to 0, this will cause the interaction to be completely omitted from force and energy calculations.

        |  |  |
        | --- | --- |
        | Parameters: |  |

        - **index** – the index of the interaction for which to get parameters
        - **particle1** – the index of the first particle involved in the interaction
        - **particle2** – the index of the second particle involved in the interaction
        - **chargeProd** – the scaled product of the atomic charges (i.e. the strength of the Coulomb interaction), measured in units of the proton charge squared
        - **sigma** – the sigma parameter of the Lennard-Jones potential (corresponding to the van der Waals radius of the particle), measured in nm
        - **epsilon** – the epsilon parameter of the Lennard-Jones potential (corresponding to the well depth of the van der Waals interaction), measured in kJ/mol

    void `createExceptionsFromBonds`(*const* std::vector<std::pair<int, int>> &*bonds*, double *coulomb14Scale*, double *lj14Scale*)¶
    :   Identify exceptions based on the molecular topology. Particles which are separated by one or two bonds are set to not interact at all, while pairs of particles separated by three bonds (known as “1-4 interactions”) have their Coulomb and Lennard-Jones interactions reduced by a fixed factor.

        |  |  |
        | --- | --- |
        | Parameters: |  |

        - **bonds** – the set of bonds based on which to construct exceptions. Each element specifies the indices of two particles that are bonded to each other.
        - **coulomb14Scale** – pairs of particles separated by three bonds will have the strength of their Coulomb interaction multiplied by this factor
        - **lj14Scale** – pairs of particles separated by three bonds will have the strength of their Lennard-Jones interaction multiplied by this factor

    bool `getUseDispersionCorrection`() *const*¶
    :   Get whether to add a contribution to the energy that approximately represents the effect of Lennard-Jones interactions beyond the cutoff distance. The energy depends on the volume of the periodic box, and is only applicable when periodic boundary conditions are used. When running simulations at constant pressure, adding this contribution can improve the quality of results.

    void `setUseDispersionCorrection`(bool *useCorrection*)¶
    :   Set whether to add a contribution to the energy that approximately represents the effect of Lennard-Jones interactions beyond the cutoff distance. The energy depends on the volume of the periodic box, and is only applicable when periodic boundary conditions are used. When running simulations at constant pressure, adding this contribution can improve the quality of results.

    int `getReciprocalSpaceForceGroup`() *const*¶
    :   Get the force group that reciprocal space interactions for Ewald or PME are included in. This allows multiple time step integrators to evaluate direct and reciprocal space interactions at different intervals: `getForceGroup()` specifies the group for direct space, and `getReciprocalSpaceForceGroup()` specifies the group for reciprocal space. If this is -1 (the default value), the same force group is used for reciprocal space as for direct space.

    void `setReciprocalSpaceForceGroup`(int *group*)¶
    :   Set the force group that reciprocal space interactions for Ewald or PME are included in. This allows multiple time step integrators to evaluate direct and reciprocal space interactions at different intervals: `setForceGroup()` specifies the group for direct space, and `setReciprocalSpaceForceGroup()` specifies the group for reciprocal space. If this is -1 (the default value), the same force group is used for reciprocal space as for direct space.

        |  |  |
        | --- | --- |
        | Parameters: |  |

        - **group** – the group index. Legal values are between 0 and 31 (inclusive), or -1 to use the same force group that is specified for direct space.

    void `updateParametersInContext`(Context &*context*)¶
    :   Update the particle and exception parameters in a `Context` to match those stored in this `Force` object. This method provides an efficient method to update certain parameters in an existing `Context` without needing to reinitialize it. Simply call `setParticleParameters()` and `setExceptionParameters()` to modify this object’s parameters, then call `updateParametersInContext()` to copy them over to the `Context`.

        This method has several limitations. The only information it updates is the parameters of particles and exceptions. All other aspects of the `Force` (the nonbonded method, the cutoff distance, etc.) are unaffected and can only be changed by reinitializing the `Context`. Furthermore, only the chargeProd, sigma, and epsilon values of an exception can be changed; the pair of particles involved in the exception cannot change. Finally, this method cannot be used to add new particles or exceptions, only to change the parameters of existing ones.

    bool `usesPeriodicBoundaryConditions`() *const*¶
    :   Returns whether or not this force makes use of periodic boundary conditions.

        |  |  |
        | --- | --- |
        | Returns: | true if force uses PBC and false otherwise |

High performance molecular simulation on GPUs


### Navigation

- Core Objects
- Forces
- Integrators
- Extras

©2015, Stanford University and the Authors.
|
Powered by Sphinx 1.3.1
& Alabaster 0.7.7
|
Page source
